# Undiagnosed Metastatic Breast Carcinoma Presenting as Thrombotic Thrombocytopenic Purpura

**DOI:** 10.7759/cureus.44452

**Published:** 2023-08-31

**Authors:** Devon L Jackson, Lamarque Coke, Olanrewaju Oni, Lekidelu Taddesse-Heath

**Affiliations:** 1 Pathology and Laboratory Medicine, Howard University Hospital, Washington, DC, USA; 2 Pathology and Laboratory Medicine, Howard University College of Medicine, Washington, DC, USA

**Keywords:** thrombocytopenia, anemia, metastatic, breast, carcinoma

## Abstract

Cancer-associated thrombotic microangiopathy has a documented relationship with metastatic disease. Other examples of thrombotic microangiopathy (TMA) include thrombotic thrombocytopenic purpura (TTP) and hemolytic uremic syndrome (HUS). All these conditions can present with microangiopathic hemolytic anemia as well as thrombocytopenia. However, when these findings occur in association with cancer, they often carry a poor prognosis. Though associated with metastasis, microangiopathic hemolytic anemia, and thrombocytopenia have rarely been seen as the presenting signs of malignancy.

We present the case of a 66-year-old female with no known history of cancer who exhibited an intriguing clinical presentation, including progressive dyspnea worsening with exertion, diarrhea, and dizziness. Laboratory investigations revealed Coombs-negative hemolytic anemia with schistocytes on blood smears and thrombocytopenia. The patient's condition raised concerns for TTP, prompting the initiation of plasmapheresis. However, despite treatment, the anemia and thrombocytopenia showed no improvement, leading to further investigations. Ultimately, a bone marrow biopsy revealed tumor cells arranged in nests and single files, leading to a diagnosis of metastatic carcinoma, consistent with breast primary. This was the patient’s first known sign of breast cancer. This case emphasizes the significance of considering metastatic cancer as a potential differential diagnosis in patients presenting with similar signs and symptoms.

## Introduction

Thrombotic thrombocytopenic purpura (TTP) is a thrombotic microangiopathy (TMA), a multisystem disorder characterized by microangiopathic hemolytic anemia (MAHA), thrombocytopenia, and ischemic manifestations caused by intraluminal platelet agglutination [1]. Other examples of TMA include hemolytic uremic syndrome (HUS) and HELLP (hemolysis with elevated liver enzyme levels and low platelets) syndrome. The essential diagnostic criteria for these disorders are MAHA and unexplained thrombocytopenia. Microangiopathic hemolytic anemia is a nonimmune hemolytic anemia marked by schistocytes, reticulocytosis, low levels of haptoglobin, and elevated levels of lactate dehydrogenase (LDH) and indirect bilirubin. These findings are due to red blood cell destruction within the microvasculature [2].

Thrombocytopenia and MAHA secondary to systemic malignancies, such as breast carcinoma, have been well described and are often referred to as cancer-associated TMA, cancer-associated TTP, or cancer-associated MAHA. Although MAHA and thrombocytopenia have been described in association with metastatic carcinomas, these are rarely the predominant and presenting clinical features in patients without initially apparent malignancies [3]. While uncommon, metastatic disease should still be kept in the differential diagnosis when evaluating patients with TTP-like features. This article aims to shed light on a rare case of undiagnosed metastatic breast carcinoma, where TTP-like clinical features led to the eventual diagnosis. This report highlights the need for early recognition and prompt intervention.

This article was previously presented as a meeting poster at the 2021 College of American Pathologists (CAP) Annual Meeting on September 26, 2021.

## Case presentation

A 66-year-old obese woman with a history of chronic obstructive pulmonary disease (COPD), diastolic congestive heart failure with preserved ejection fraction, and coronary artery disease presented with shortness of breath and diarrhea. Her dyspnea had progressively worsened over three to four months, worsening with exertion. She had associated dizziness and lightheadedness. An initial complete blood count showed anemia and thrombocytopenia with decreased hemoglobin, hematocrit, and platelet count. A peripheral blood smear then revealed numerous schistocytes, moderate spur cells, and 35 nucleated red blood cells per 100 white blood cells. Additional laboratory investigations were consistent with hemolytic anemia, showing a significantly increased serum lactate dehydrogenase, mildly increased total bilirubin, increased direct bilirubin, and decreased haptoglobin. A basic metabolic panel raised concern for renal dysfunction with increased creatinine and blood urea nitrogen. A coagulation panel revealed a mildly prolonged prothrombin time and increased D-dimer, while partial thromboplastin time, fibrinogen, and fibrin degradation product levels were all normal (Table 1).

**Table 1:** Laboratory data of the patient

Variable	Result	Reference range
Hemoglobin (g/dL)	8.6	12.1 - 15.9
Hematocrit (%)	27.8	34.3 - 46.6
Platelets (x10^9^/L)	51	177 - 406
Lactate dehydrogenase (IU/L)	790	100 - 250
Haptoglobin (mg/dL)	<30	36 - 195
Total bilirubin (mg/dL)	1.3	0.2 - 1.2
Direct bilirubin (mg/dL)	0.5	0 - 0.2
Creatinine (mg/dL)	2.63	0.6 - 1.1
Blood urea nitrogen (mg/dL)	28	7 - 25
Prothrombin time (sec)	14.6	12.5 - 14.5
Partial thromboplastin time (sec)	32	25 - 35
D-dimer (μg/mL)	2.73	0 - 0.48
Fibrinogen (mg/dL)	293	237 - 507
Fibrin degradation products (μg/mL)	<10	<10
ADAMTS13 activity (%)	87	68 - 163

A direct Coombs test was negative. These laboratory and peripheral smear findings raised concern for TTP. The ADAMTS13 (a protease enzyme responsible for cleaving von Willebrand-factor (vWF) multimers) levels were obtained, and the patient was started on plasmapheresis.

**Figure 1:**
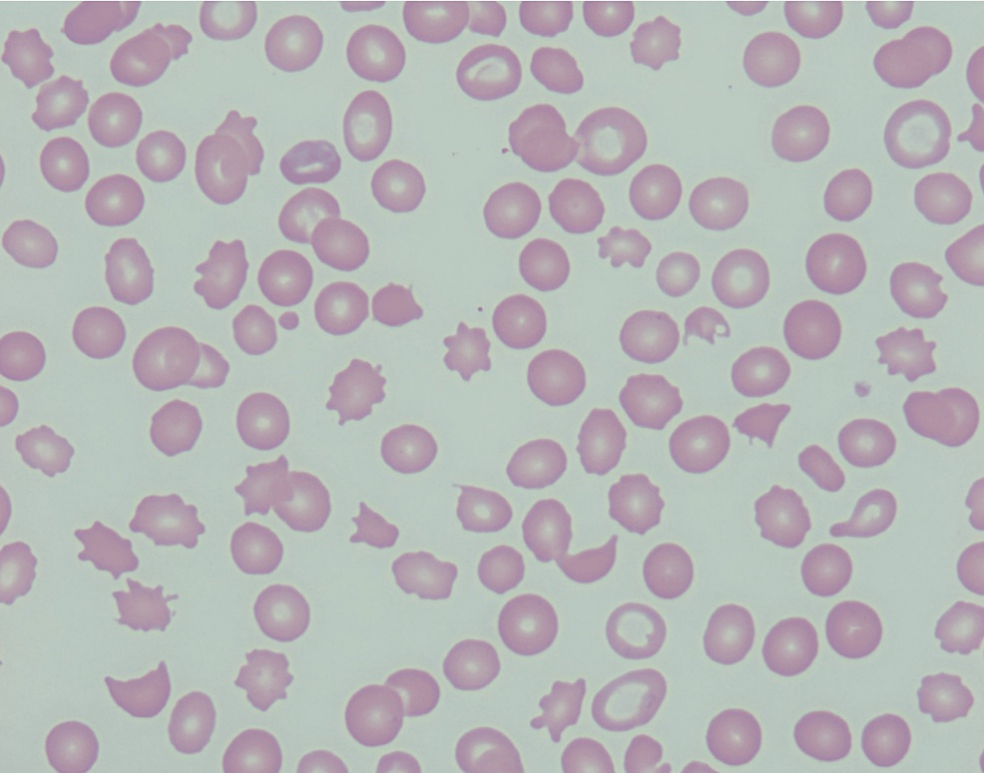
A peripheral blood smear shows anisopoikilocytosis, numerous schistocytes, polychromasia, and reduced platelet count (1000x).

**Figure 2:**
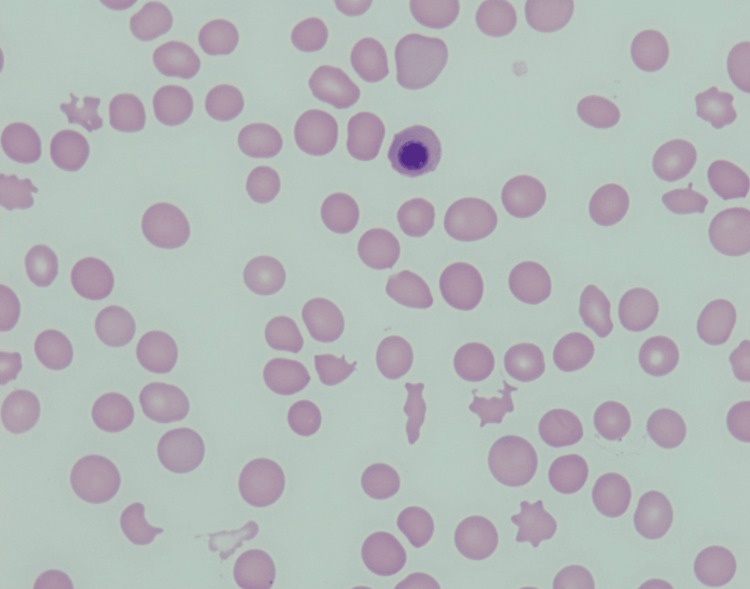
A peripheral blood smear shows nucleated red blood cells with multiple schistocytes and a reduced platelet count (1000x).

Despite undergoing plasmapheresis over five days, the patient’s anemia and thrombocytopenia showed no improvement. The ADAMTS13 level was within normal limits (Table 1). Given her persistent bicytopenia, a bone marrow biopsy was recommended. A repeat peripheral smear revealed similar findings as the initial evaluation, with the persistence of the thrombocytopenia, numerous schistocytes and fragmented red blood cells (RBCs), polychromasia, and nucleated RBCs with marked anisocytosis and poikilocytosis (Figures 1, 2).

**Figure 3:**
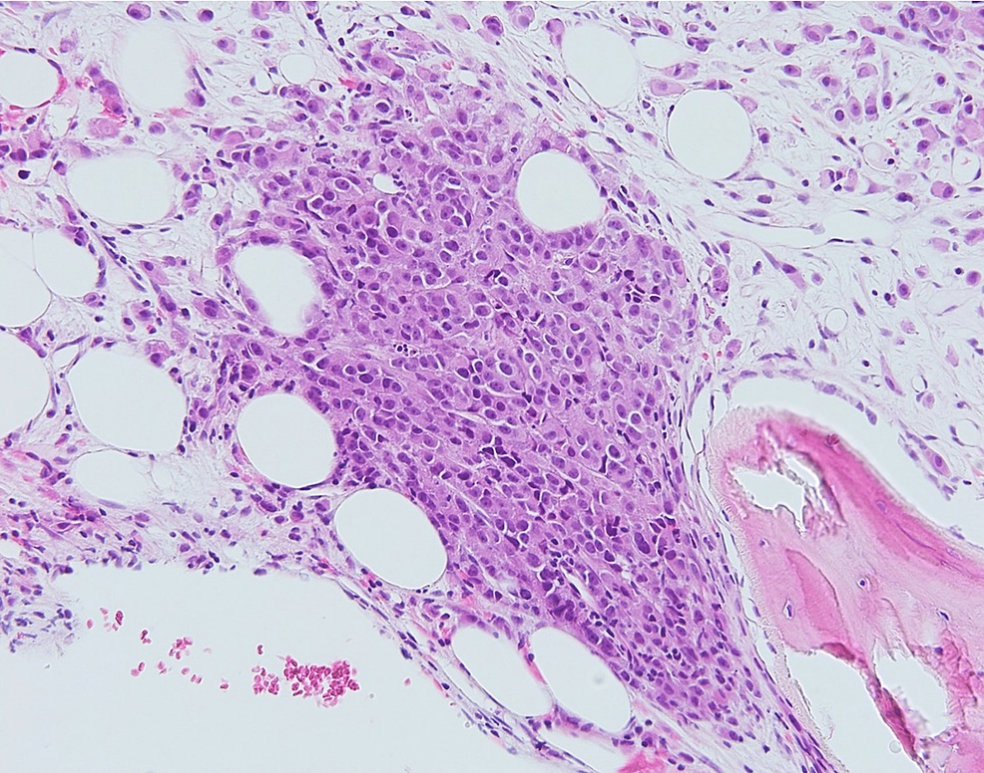
A bone marrow core biopsy shows infiltration by sheets, nests, and single tumor cells with associated fibrosis (200x, H&E).

**Figure 4:**
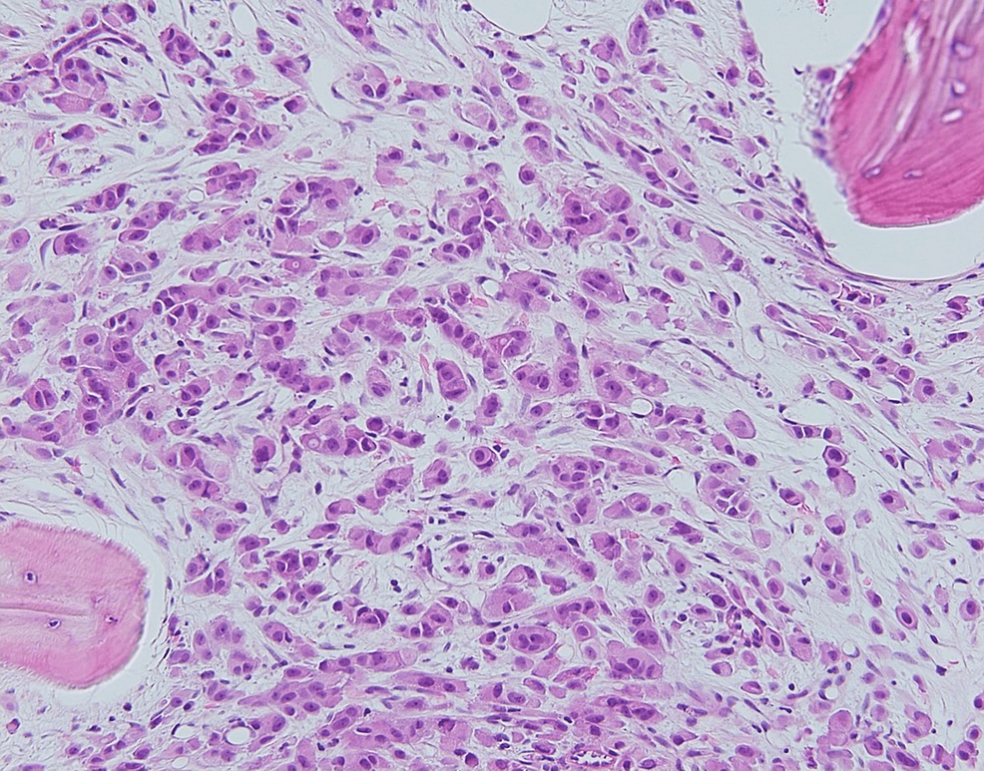
A bone marrow core biopsy shows infiltration by sheets, nests, and single tumor cells with associated fibrosis (200x, H&E).

A bone marrow biopsy was performed and showed extensive replacement of the marrow by nests of tumor cells and individual epithelial cells arranged in a single file in a background of fibrosis, consistent with metastasis (Figures 3, 4).

The marrow showed only a focal area with maturing myeloid and erythroid cells with few megakaryocytes.

**Figure 5:**
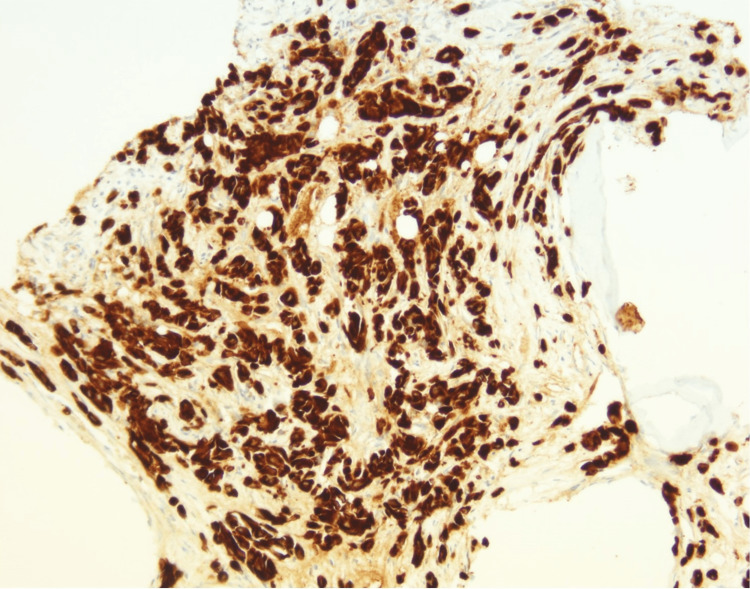
Immunohistochemistry of the bone marrow core biopsy shows tumor cells staining positively with pancytokeratin (200x).

**Figure 6:**
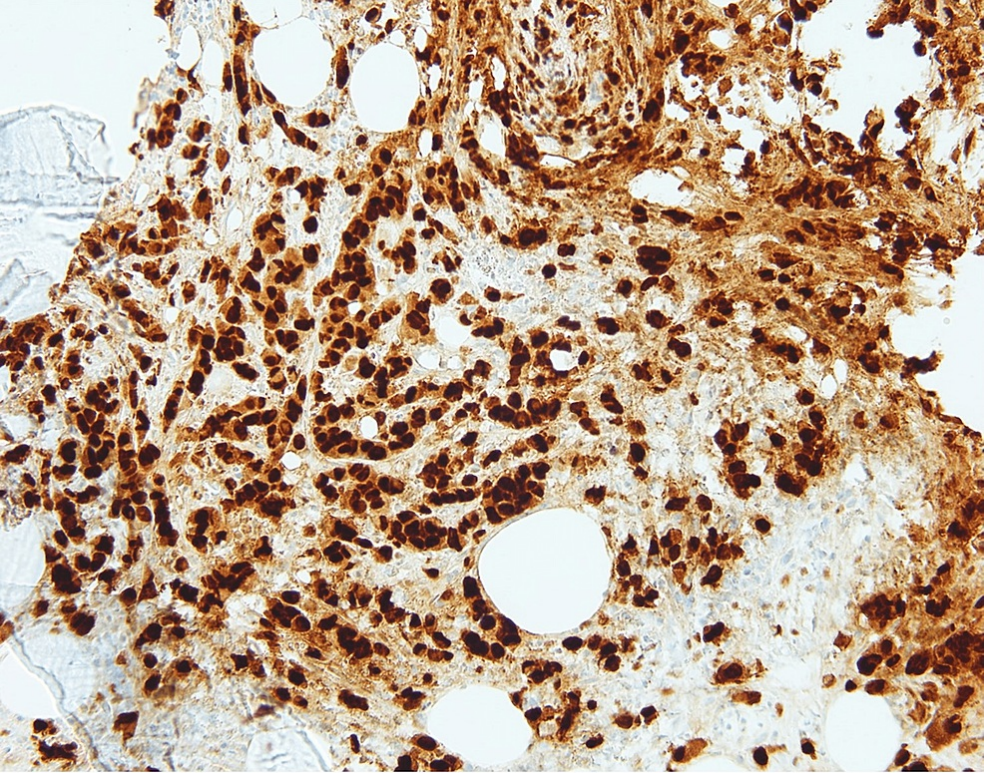
Immunohistochemistry of the bone marrow core biopsy shows tumor cells staining positively with GATA-3 (200x). GATA-3: GATA binding protein 3

**Figure 7:**
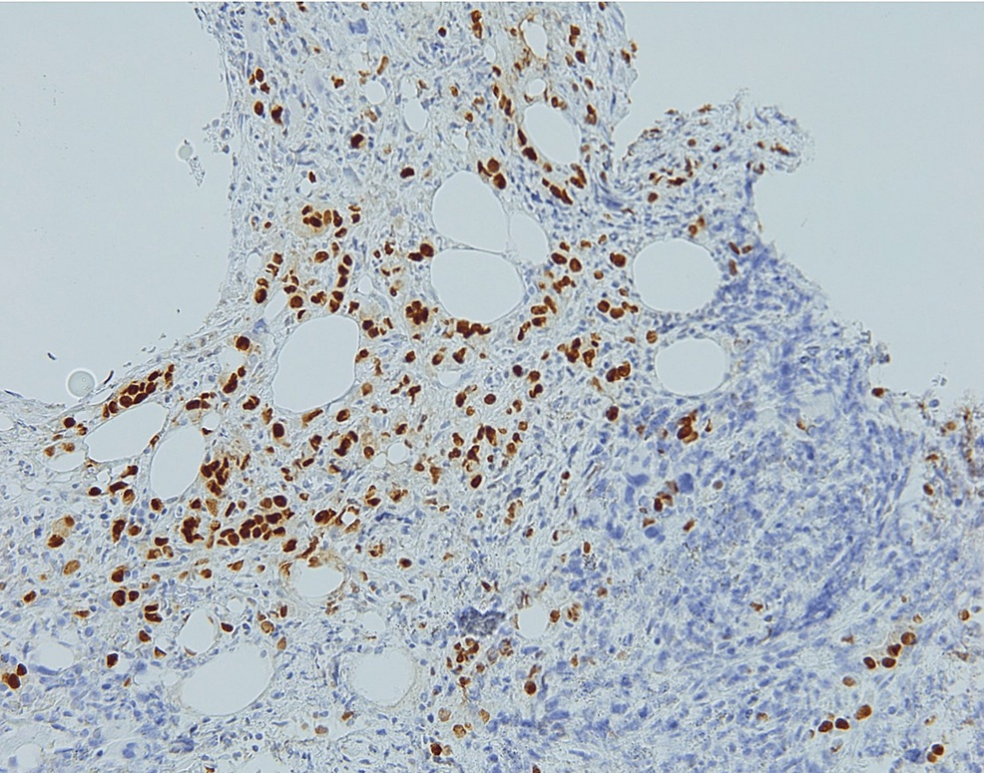
Immunohistochemistry of the bone marrow core biopsy shows tumor cells staining positively with the estrogen receptor (200x).

The tumor cells in the marrow were positive for pancytokeratin (Figure 5), GATA binding protein 3 (GATA-3) (Figure 6), estrogen receptor (Figure 7), and focally positive for mammaglobin and gross cystic disease fluid protein 15 (GCDFP-15).

**Figure 8:**
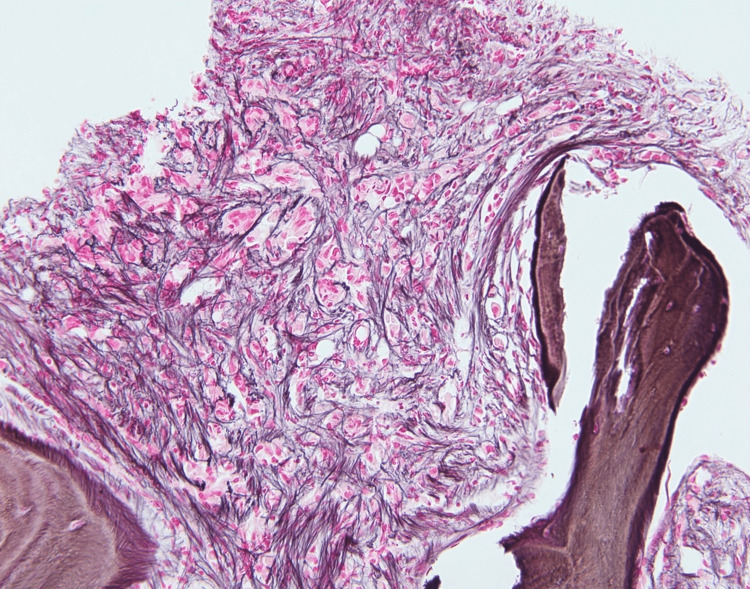
A special stain of the bone marrow core biopsy; a reticulin stain showing a marked increase in reticulin fibrosis associated with the tumor cells (200x).

They were negative for cytokeratin 7 (CK7), cytokeratin 20 (CK20), thyroid transcription factor 1 (TTF-1), paired-box gene 8 (PAX8), and uroplakin. A reticulin stain showed a marked increase in reticulin fibrosis (Figure 8).

The immunohistochemical profile was consistent with metastatic carcinoma, breast primary. Cytokeratin 7 was unexpectedly negative; however, this may be due to the decalcification of the specimen. Following the bone marrow findings, a nuclear whole-body bone scan was performed and showed no definitive evidence of a primary tumor or metastasis at other sites. The patient wanted to continue her care at her primary care institution, so she was discharged once stable. After being discharged from our hospital, the patient required admission to another hospital for ongoing gastrointestinal (GI) complaints. She had not started any treatment for her breast cancer due to her GI issues, and her clinical condition worsened. She went into rapidly progressive shock and subsequently expired, three weeks after the diagnosis of metastatic carcinoma was established.

## Discussion

Cancer-related MAHA and thrombocytopenia are serious complications of malignancies and are associated with a poor prognosis, often leading to rapid mortality within a short time of diagnosis, with or without treatment [1,4]. The prevalence of cancer-related MAHA ranges from 3.5% to 7.8%, with an estimated incidence of approximately 0.25 to 0.45 persons per million [4]. A relatively high proportion of cases occur at the time of cancer recurrence. While these complications can be diagnosed concurrently with the initial cancer diagnosis, they are rarely observed as the primary manifestations of a malignancy [4,5]. The most common malignancies associated with cancer-related MAHA and thrombocytopenia are adenocarcinomas, specifically gastric, breast, prostate, and lung [4,5]. Among breast cancer cases, lobular histology is frequently documented [6], but ductal and colloid types have also been reported [2,7-9]. In our case, the bone marrow findings revealed tumor cells arranged in a single-file pattern, suggesting lobular histology, although confirmation awaited a possible biopsy of the primary tumor. Regardless of the type of cancer, early recognition of MAHA and thrombocytopenia in association with cancer is critical to initiating appropriate treatment promptly. The diagnosis of cancer-related MAHA can occur two to 14 days after an initial diagnosis of TTP, with a median of six days [3].

Diagnosing cancer-related MAHA and thrombocytopenia presents challenges as they require distinguishing them from other TMA disorders like TTP and HUS. Classically, HUS consists of renal failure, thrombocytopenia, and MAHA, while TTP consists of fever, neurologic symptoms, renal failure, MAHA, and thrombocytopenia [9]. Microangiopathic hemolytic anemia is typically characterized by a negative direct Coombs test, reticulocytosis, schistocytes, elevated levels of LDH and indirect bilirubin, and low levels of haptoglobin [2]. Several features favor cancer-related MAHA and thrombocytopenia, including older age at presentation (usually mid-50s versus early-40s in TTP), presenting complaints of back pain and dyspnea, ADAMTS13 activity generally closer to 50% (versus <5%-10% typically seen in TTP), lack of response to plasmapheresis, and evidence of active or recurrent malignancy [9]. An additional key feature is significantly elevated serum LDH in patients with malignancy, suggesting tumor lysis [3,5]. These findings in our case made the diagnosis of TTP less likely and prompted a bone marrow biopsy to investigate the persistent bicytopenia.

The detection of new or recurrent malignancy in cancer-related MAHA and thrombocytopenia is usually through the involvement of the bone marrow, as bone marrow is the most common site of metastasis seen in these cases [4,9]. The degree of involvement can vary, ranging from moderate to focal, and in some instances, the bone marrow may not be involved at all [4]. Imaging, such as computed tomography or a bone scan, can aid in the diagnosis of systemic malignancy [3]. In our patient, a bone scan did not contribute to the confirmation of metastatic disease. A biopsy of the bone marrow has proven most beneficial for diagnosis in most cases [3]. When the marrow is involved, clusters of malignant epithelial cells or single epithelial cells may be seen, particularly in breast cancer cases [7,8]. Similar findings were observed in the bone marrow biopsy in our case. The pathophysiology of cancer-related MAHA and thrombocytopenia is not yet fully understood, but many theories exist based on what is known regarding TTP. Consistent findings in TTP are the deficiency or inhibitor of ADAMTS13, a von Willebrand factor (vWF)-cleaving protease leading to the presence of unusually large (uL) vWF multimers in the serum; ADAMTS13 cleaves vWF multimers to prevent platelet activation and aggregation. However, when this cleavage process is impaired, the multimers can be released into the circulation, leading to MAHA and thrombocytopenia [10].

In contrast, cancer-related MAHA and thrombocytopenia are thought to involve increased angiogenesis in bone marrow for the growth of the cancer. Abnormal angiogenesis, tumor growth, and secondary myelofibrosis may then cause endothelial cell injury by direct vessel involvement [10]. Fibrosis, although not specific, has been described in many cases [4]. Increased fibrosis was a major feature seen in our case. Significant fibrosis and encroachment of the tumor on vessels could cause the release of uLvWF multimers. With a possible decrease in the activity of ADAMTS13 due to autoreactivity triggered by the tumor, platelets aggregate in vessels, again leading to MAHA and thrombocytopenia [10,11]. While ADAMTS13 activity may be reduced in cancer patients, it is not as low as that seen in TTP [4]. In fact, levels may even be normal, as in our case. Another potential mechanism that has been proposed for the changes in cancer patients is related to the type of cancer. Since adenocarcinomas are the most common cancers identified in these cases, it is speculated that mucin production may cause a direct effect on the endothelial cells, leading to detrimental changes in endothelial function [10].

Given the significant vessel involvement in these cases, disseminated intravascular coagulation (DIC) may be seen. Disseminated intravascular coagulation is highly associated with cancer-related MAHA and thrombocytopenia, but it rarely occurs in primary TTP [4,11]. Disseminated intravascular coagulation was not diagnosed in our case, as some coagulation parameters were normal. Even without DIC, as we saw in this case, the outcome can be fatal. Urgent treatment with exchange plasmapheresis is critical when TTP is suspected, as it is the standard treatment, but the response to this treatment is limited in cancer-related MAHA and thrombocytopenia. Exchange plasmapheresis may even be harmful in patients with malignancies [5]. The ultimate treatment is systemic chemotherapy. In one series, 46.5% of patients died from cancer-related MAHA within one month, with or without treatment [4]. Despite the advanced stage, patients with metastatic breast cancer were found to have longer overall survival than patients with other metastatic malignancies [12]. Compared to breast cancer patients without MAHA, the overall survival of breast cancer patients with MAHA is inferior, even with chemotherapy. The overall survival for breast cancer patients without MAHA treated with chemotherapy ranges from 11 to 33.5 months, versus four months for breast cancer patients with MAHA treated with chemotherapy [4].

While the prognosis is generally poor, initiating chemotherapy is the only reliable treatment option for cancer-related MAHA and thrombocytopenia and requires arriving at a diagnosis in a timely manner [5]. Therefore, it is imperative to have a high index of suspicion for an underlying malignancy when a patient presents with MAHA and thrombocytopenia.

## Conclusions

Microangiopathic hemolytic anemia (MAHA) and thrombocytopenia as the initial manifestations of a malignancy are rare occurrences. Our case highlights TTP-like clinical features as the initial presentation of undiagnosed metastatic carcinoma. Since the patient had no history of malignancy, metastatic carcinoma was not initially considered the underlying cause of her MAHA and thrombocytopenia. This case illustrates the need to include cancer in the differential diagnosis at the time of presentation. Given the overlapping clinical features with TTP, it is advisable to initiate exchange plasmapheresis. However, if there is little to no response, further workup is warranted to look for metastatic disease. Once metastatic disease is confirmed, appropriate treatment can be started. Prognosis is generally poor in most cancer-related cases of MAHA and thrombocytopenia, but initiating chemotherapy offers the best chance for these patients in terms of management and potential outcomes.
